# Self-organizing pattern of subpleural alveolar ducts

**DOI:** 10.1038/s41598-020-59752-3

**Published:** 2020-02-21

**Authors:** Wayne Mitzner, Jeffrey Loube, Jarrett Venezia, Alan Scott

**Affiliations:** Johns Hopkins University, Baltimore, Maryland US

**Keywords:** Physiology, Anatomy

## Abstract

In this study we have utilized an optical clearing method to allow visualization of a heretofore undescribed subpleural acinar structural organization in the mammalian lung. The clearing method enables visualization of the lung structure deep below the visceral pleura in intact inflated lungs. In addition to confirming previous observations that the immediate subpleural alveoli are uniform in appearance, we document for the first time that the subpleural lung parenchyma is much more uniformly organized than the internal parenchyma. Specifically, we report that below the surface layer of alveoli, there is a striking parallel arrangement of alveolar ducts that all run perpendicular to the visceral pleural surface. A three dimensional visualization of alveolar ducts allowed for a calculation of the average inner to outer duct diameter ratio of 0.53 in these subpleural ducts. This unique, self-organizing parallel duct structure likely impacts both elastic recoil and the transmission of tethering forces in healthy and diseased lungs.

## Introduction

Alveoli in the region just below the pleural surface have been examined by light microscopy since the 1930s^1–7^. These studies have often examined how the alveoli expand with lung inflation and how they might change with lung pathology. However, because light scattering from alveolar walls limits the depth of visualization with conventional light microscopy, there has always been some question as to whether this subpleural alveolar anatomy is representative of the structure throughout the lung. On the one hand the relatively thick visceral pleura forms a fixed boundary to one facet of an alveolus, and this must offer a level of constraint that is not present in internal alveoli. On the other hand, there are on the order of a dozen or so facets of an alveolus that are not constrained by being connected to the relatively thick pleura, so the degree to which the presence of a pleural boundary affects alveolar structure is not well understood. That there might be a difference in structural organization near the pleura was shown by a study where the mean airspace chord length was shown to be smaller when assessed closer to the visceral pleura^8^. Although it is clear from light microcopy that there are alveoli immediately under the pleural surface, there has been no information of how the alveolar duct might be oriented in this region. Traditionally, the terminal airways (respiratory bronchioles and alveolar ducts) are assumed to be somewhat randomly oriented in the lung. However, a random orientation would predict that a certain percentage of the ducts would run parallel to the pleural surface, a situation that would seem to be structurally and mechanically untenable.

In the present work, we sought to address this anatomical issue by examining the subpleural terminal lung structure using confocal fluorescent microscopy of optically cleared lungs. The optical clearing method permits imaging in the mouse lung to depths exceeding 30 alveolar diameters into the lung, allowing optical serial sectioning of the subpleural alveoli and the ducts beneath them. Our studies were initially focused on mouse lungs, but since the distinctive organization we found had not been described, we confirmed in other species that this pattern was not unique to the mouse. Results in all species show that all of the most peripheral alveolar ducts are oriented perpendicular to the visceral pleural surface.

## Methods

Intact lungs were isolated from four male BALB/cJ mice (Jackson Labs), 8–12 weeks of age. All protocols were approved by the Institutional Animal Care and Use Committee of the Johns Hopkins University. The experiments were conducted under the *Guidelines for Care and Use of Laboratory Animals* issued by the USA National Institutes of Health. Briefly, mice were sacrificed with an anesthetic overdose, and within one minute after they stopped breathing, the thorax was surgically opened. Lungs were perfused slowly through the right ventricle with 10 mL of PBS to remove red blood cells from the vasculature. The trachea was then cannulated and the lungs inflated *in situ* with 10% buffered formalin at a distending pressure of 25 cmH_2_O for at least 10 minutes. While the pressure was maintained, the trachea was then tied off and the fully inflated whole lung was removed and submerged in the same fixative for 24–48 hours prior to proceeding to the clearing protocol.

### Clearing

To clear the lungs we followed the approach described by Li, *et al*.^9^, with minor modifications. Briefly, the fixed lungs were immersed in ≈2 mL of an aqueous-based tissue clearing solution that was prepared by dissolving Histodenz (Sigma-Aldrich) at 1.45 g/ml of a buffer solution of 40% (V/V) N-methylacetamide (Sigma-Aldrich) containing 0.1% Triton X-100 and 0.5% 1-thioglycerol^9^. Glass containers with the individual inflated lobes were placed on a continuous rocker in the dark for at least 1 week. Tissues were checked every few days, and if the clearing process slowed, fresh clearing media was added. In four additional similarly treated lungs we compared the volumes before and after 2 weeks of immersion in the clearing media. There was only minor nonsignificant volume shrinkage (9.3% - equivalent to 3.3% linear shrinkage) and no gross tissue distortion of the lung during this clearing procedure.

### Microscopy

Cleared lungs were placed in an Attofluor cell chamber (Thermo Fisher Scientific) filled with the tissue clearing solution described above and imaged with a Zeiss LSM 880-Airyscan FAST laser scanning confocal microscope. A 10x magnification was used for a majority of the image sequences, acquired using a 561 nm laser at 100% power. At this magnification, the xyz resolution created a voxel size of 1.38 × 1.38 × 10.0 µm. The imaging process for the whole mouse lung involved taking multiple stacks in the z direction (called z stacks, (1024×1024 pixels or 1.41 × 1.41 mm in x and y), and then stitching these together to get the whole lung images. The time for obtaining the entire 3D image sequence of an entire lobe of a mouse lung (≈15 z stacks) was approximately 90 minutes. For select areas of the lung we used the highest sampling frequency to visualize the maximal resolution of the system. This resulted in a slice spacing of 3.4 µm, but the imaging acquisition time proved to be prohibitively long for imaging the entire lung at this higher resolution (≈ 40 min for a single z-stack). In all images reported here, lung structure was visualized by taking advantage of the intrinsic autofluorescence from cells and extracellular matrix of lung tissue^10–12^.

## Results

Confocal imaging of intact, cleared mouse lungs allowed us to image the structure at depths that approached 1500 µm below the pleural surface. There is of course still a limitation to the depth of imaging even with the cleared lungs, since some light is reflected from the multiple alveolar walls resulting in decreased signal and increased noise the deeper you sample. However, the ability to visualize structures in mice up to 30–40 alveolar diameters below the pleura provides a unique opportunity to understand how this region of the lung is structured and organized. A typical sequence of serial sections from a left lung is shown in supplemental Video 1. Shown are 84 sections taken every 10 µm, starting from the pleural surface to a depth of 840 µm. The time for obtaining the entire 3D image sequence of the whole lobe (≈15 z stacks) was approximately 90 min. At increasing depth, duct branching can be seen as well as the distal ends of small pulmonary blood vessels. In supplemental Video 2, a single z stack was done with the higher resolution in the z direction providing the minimal z spacing of 3.4 µm. This particular stack includes 243 images for a total subpleural depth of 833 µm. As with the 10 µm sections, at depths more than 2 alveoli deep, the parallel organization of the alveolar ducts is evident as well as the intimate relationship between the basolateral aspects of the alveoli surrounding adjacent ducts. This stack also affords a higher resolution imaging of the branching structures of the blood vessels (bright autofluorescence) and ducts in the deeper lung. This highest resolution, however, was not routinely utilized because the imaging took much too long.

**Figure 1:**
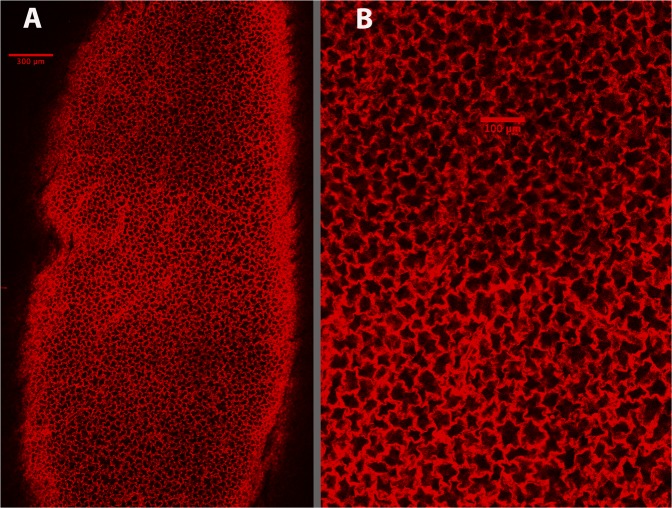
Low (**A**) and high (**B**) magnification images of the subpleural alveoli visualized in a cleared mouse lung at 20 µm below the pleural surface.

Taking 10 µm optical slices parallel to the pleura revealed a uniform layer of alveoli immediately below the pleural surface, as previously reported^2,13^. Figure 1A shows a typical low power image of the subpleural alveoli at 20 µm below the pleura. Figure 1B shows these subpleural alveoli at higher magnification. We obtained a rough estimation of the size of the alveoli in this initial subpleural layer by overlaying an image with a point grid to randomly select 50 alveoli/lung. The luminal areas of the alveoli so selected were measured using ImageJ. For a more intuitive insight, this area (A) was converted to an equivalent diameter as the square root of (4 A/π). We found the average (±SD) diameter of the subpleural alveoli in cleared BALB/cJ lungs to be 37.6 ± 10.3 µm.

**Figure 2:**
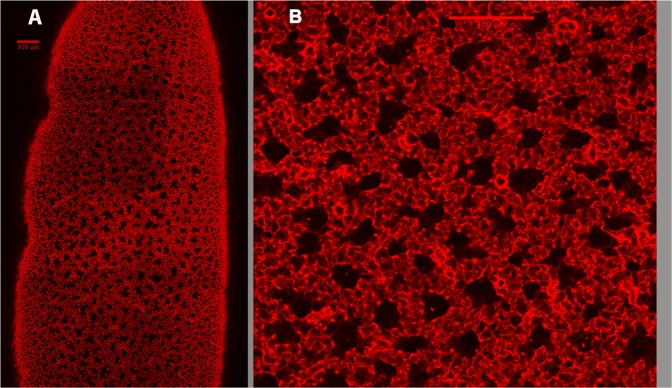
Low (**A**) and high (**B**) power images of mouse alveolar ducts visualized 100 µm below the visceral pleura. All the black holes in the low power image are duct lumens. (The varying light and dark square regions in the low power image (2A) result from a reconstruction artifact in stitching the separately imaged regional stacks.) In 2(**B**), where there are adjacent ducts, there is a clear double layer of alveoli between them.

Importantly, as one focuses progressively deeper into the lung beyond this initial subpleural alveolar layer, there emerges a very striking subpleural structural organization consisting of multiple parallel alveolar ducts oriented perpendicular to the pleural surface. Figure 2A shows a low power mouse lung image illustrating a large number of adjacent alveolar ducts in cross-section. The average depth below the pleural surface in this image in Fig. 2A is 100 µm (about 3 mouse alveolar diameters).

**Figure 3:**
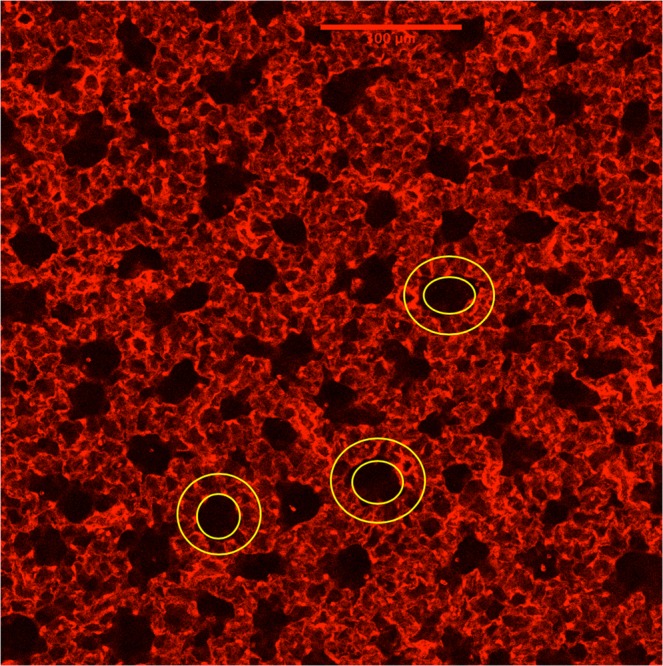
Image showing how a rough estimate of the subpleural duct dimensions was made. Ovals were placed in lumen of circular appearing ducts. A second circle was drawn around the outer border of the alveoli that surround this lumen.

Figure 2B shows a higher magnification image of these mouse lung ducts. Duct outer walls are shared with adjacent ducts, and where they touch results in a common wall consisting of a double alveolar layer. Although many ducts appear cylindrical, there is no easy objective way to quantify these duct structures. We nevertheless attempted to obtain an estimate of both the inner and outer borders of the ducts. Since there is no physical border in the duct lumen or outer boundary, we could not use ImageJ to calculate the area as was done for the subpleural alveoli. Therefore, as an initial somewhat biased approach, circles were fit by eye to the outer and inner boundaries of randomly selected ducts that had a round shape. An example of what was done is shown in Fig. 3. At least 30 ducts were analyzed in each animal. The average (±SD) outer diameter of the duct in this strain of mouse was 159.5 ± 37.8 µm and the inner lumen was 81.6 ± 20.1 µm, resulting in an inner to outer ratio of 0.51 ± 0.03.

## Discussion

We have employed a recently described approach to clear lung tissue^9^ to allow confocal microscopic imaging to depths up to 1500 µm below the visceral pleural surface in the mouse. In preliminary testing we compared lung tissues processed with the clearing method to intact lungs that were simply fixed but not cleared. We found for the uncleared tissue that, while the initial surface layer of alveoli appeared similar to that of the cleared lungs, with confocal imaging beyond a depth of about ≈60 µm it was impossible to clearly define any of the lung structures. At a depth of 90 µm, the image resolution in uncleared lungs became increasingly worse, with higher optical noise and very poor resolution of the unique duct structure so obvious in the cleared lungs. A different clearing method was described by Li *et al*.^14^, and though they were mainly interested in brain tissue, they tested the method in fixed lungs. Unfortunately they gave no information on how the lungs were fixed, and the few lung pictures shown were from a 1 mm internal section of the lung, so alveolar ducts were not easily recognizable. Scott, *et al*.^15^ also described a clearing method and used this in mice to trace pulmonary nerves in 3 dimensions. These authors showed some beautiful images of segmented airway trees, and though they did not look at the subpleural acinar structure, the parallel duct structure we show in the present study is very consistent with the orientation of the terminal bronchi shown by Scott, *et al*.

**Figure 4:**
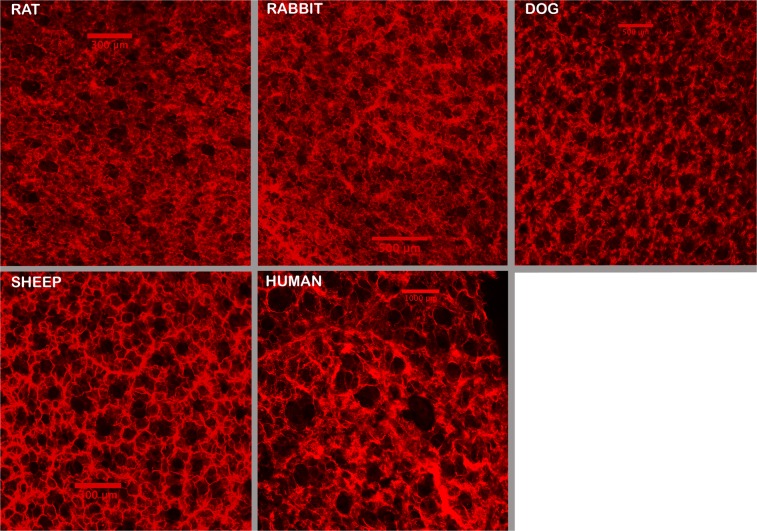
Images from 5 larger species showing the presence of subpleural alveolar ducts as were found in the mice. The rabbit and rat lungs were prepared as in the mouse, but the dog, sheep, and human lungs were not perfused to wash out the blood. For these larger lungs, after the lungs were fixed for at least 2 weeks with formalin, small mouse lung size pieces were cut and placed in the clearing solution. When cleared, they were similarly imaged through the visceral pleural surface. Image depth in the larger species becomes limited very quickly, but in all species, parallel perpendicular ducts are readily seen below the initial subpleural layer of alveoli. Sources of animals: male adult Sprague Dawley rat; male adult NZ White rabbit; male mongrel 20 kg dog; male 25 kg sheep; tissue from 1 human (61 y/o female). The human lung tissue was supplied from another investigator and the lung was not inflated for fixation, as done in all the other species.

We initially used this clearing and imaging approach only in mice, with the long-term goal of studying structural changes in emphysema. However, after seeing the unexpected parallel organization of subpleural alveolar ducts, we then wondered if this parallel organization of alveolar ducts was unique to the mouse. To this end we obtained, from other investigators’ protocols, postmortem lungs from several larger species (rat, rabbit, dog, and sheep, and a deidentified human lung sample), and prepared the tissues for clearing and imaging in the same manner as outlined for the mouse. All species studied showed the same parallel stacking of subpleural alveolar ducts in an orientation that was perpendicular to the pleural surface (Fig. 4). For species with larger alveoli, the ability the ability to visualize structures deep below the initial pleural alveolar layer becomes increasingly limited. In the extreme case of the human lung where the alveolar diameter is about 10 times larger than the mouse, the depth of imaging only allowed for visualization of the initial segments of the alveolar ducts.

With regard to alveolar volume or size, the only way to measure this accurately is by using a disector probe to determine the number of alveoli and then dividing this into the total alveolar volume^16,17^. This procedure is complex and not commonly performed. On a normal histologic section through the interior of the lung, alveolar size can’t be measured, since the section will cut alveoli at random orientations, sometimes just sampling an edge and sometimes going through the middle. Even defining what is meant by alveolar size is controversial. However, when viewed through the pleural surface, individual alveoli are uniformly spread across the surface, and this allows an estimate of the size of this unique subpopulation of alveoli. Our approach of measuring the internal area of randomly selected alveoli and then calculating an equivalent diameter provides an intuitive metric. One limitation of this method results from the fact that, while the confocal imaging plane is perfectly flat, the pleural surface is not. Thus individual alveoli are not imaged on the same plane below the pleural surface. Nevertheless, the estimated average “diameter” that we report here is quite reasonable based on commonly reported values of alveolar chord length from histologic analyses. In 1963 Tenney and Remmers^13^ published a morphologic allometric study of lung structure in 26 mammalian species. In this study they air dried lungs under pressure, then quantified the subpleural alveolar “diameters” by manual measurements of images from serial histologic sections. For the mouse, they reported a value of 40 µm, which is close to our average of 37.6 µm. In contrast, Mercer *et al*. estimated an alveolar size of 58 µm by reconstructing a limited number of alveoli from serial histologic sections^18^. This larger estimate of diameter may have been influenced by the fixation and processing of the lung tissue and the fact that the alveoli selected for reconstruction were from the interior of the lung, with a wide opening into an alveolar duct, making calculation of an equivalent diameter somewhat ambiguous.

The appearance of parallel packed columns of alveolar ducts running perpendicular to the visceral pleural surface was at first quite unexpected. To our knowledge, this is the first recognition and report of this distinctive orientation of these sub-pleural alveolar ducts. However, with hindsight the configuration makes some intuitive sense if one tries to imagine alternative orientations in the context of constraints imposed by a fixed pleural boundary layer. Once the optical sections were below the initial alveolar layer, there were always adjacently stacked alveolar ducts visualized. In the mouse with its small alveoli and ducts, we were able to continue to image deeper to the point where we could visualize ducts bifurcating and the branching of small terminal bronchioles and blood vessels (see the serial sections in Videos 1 and 2). As expected, these deeper structures no longer were all oriented perfectly perpendicular to the pleura, and if we could have gone much deeper, it is likely the confocal sections would have begun to look more like the random orientation observed in conventional histologic sections.

**Figure 5:**
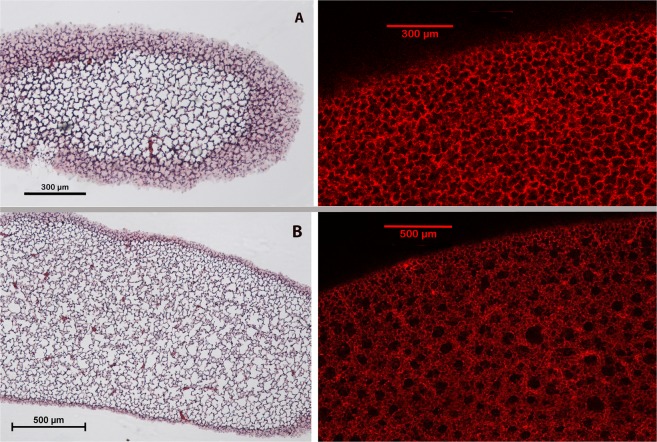
Comparison of confocal and histologic images at 20 µm (**A**) and 80 µm (**B**) below the visceral pleura. Subpleural alveoli are clearly seen in both the intact and histologic sections at 20 µm, but the duct structure below this initial level is only apparent in the intact lung.

There is another factor, however, that may play an even larger role in the different appearances between what we have observed below the pleura in the intact lungs and conventional histologic sections. In an attempt to see if we could duplicate what we found in intact cleared lungs with histologic sections, we fixed a healthy mouse lung with the identical procedure as used for clearing. Instead of clearing, however, we then subjected the lung to the most common histologic procedures of dehydration and embedding in paraffin. Before embedding, whole lobes were placed on their pleural surfaces so the sections would be cut parallel to the pleura. As with the confocal images, we cut 10 µm sections and compared these to the confocal images. Figure 5 shows a comparison of sections cut 20 µm and 80 µm below the pleura. Figure 5A shows that the subpleural alveolar layer (20 µm) appears very comparable with both methods. In fact the average (±SD) estimate of the subpleural alveolar diameter from the histologic sections was 34.5 (±3.9) µm, which is close to that from the intact cleared lung (37.6 µm). However, as one images deeper (Fig. 5B), the pattern of parallel alveolar ducts is not at all apparent in the histologic sections. Although Fig. 5B only shows one slice at a depth of 80 µm, this absence of obvious duct structure was true regardless of the depth at which the slices were cut. It seems that the conventional processes of dehydration, embedding, and sectioning distort the subpleural acinar structure sufficiently to make it impossible to see the normal 3D parallel duct organization present in intact lungs. We don’t know if less common procedures such as perfusion fixation and/or methacrylate embedding would have better preserved the normal *in vivo* structure, but this structural comparison in Fig. 5 does raise questions about the level of structural distortion with conventional procedures even deeper in the lung.

In considering how the lung is constructed, it has long been known that when the lung is inflated, all the airways lengthen^19,20^. The fact that all the ducts bordering the pleural surface are running perpendicular means that all of these ducts must also be stretched in accordion-like fashion as the lung inflates. In the mechanical model of the alveolar duct proposed by Wilson and Bachofen^21^, there are coiled structural fibers supporting the lumen and outer border of the duct. However, with inflation, these coiled fibers that attach to the pleural surface are all stretched and served to increase tension throughout the parenchyma. This consistent perpendicular structural orientation of the most peripheral ducts likely impacts the lung elasticity as well as the manifestation of emphysema, but how this would occur remains to be determined.

Finally, we note that the spatial results we have observed in adult lungs may provide unique insights into the self-organized pattern formation processes during lung development. The signaling mechanisms that promote this self-organized development may be related to activation of cell surface receptors as was recently shown^22^, although the *in vivo* situation with lung development in three dimensions is clearly more complex. Indeed, the mechanisms of this patterning process described by Su, *et al*.^22^ is likely similar to what occurs inside the lungs, except that the pleural surface plays a role in the signaling gradients guiding cell location and fate.

In summary, we have utilized a 3D optical clearing method to allow visualization of subpleural acinar structure. Consistent with the literature, we have shown a uniform layer of alveoli just below the visceral pleura, but more importantly, we have documented that the subpleural lung parenchyma is much more uniformly structured than the internal parenchyma. Specifically, we have shown that the most peripheral alveolar ducts appear to self-organize to run perpendicular to the visceral pleural surface.

## Supplementary information

Supplemental info for videos.

Video 1.This is a sequence of confocal 84 confocal sections below the visceral pleura in an optically cleared mouse left lung. Confocal sections are spaced at 10 µm intervals.

Video 2.This is a single z-stack (1.4x1.4 mm) of 243 confocal sections below the visceral pleura in an optically cleared mouse lung. Confocal sections are spaced at 3.4 µm intervals.
