# Supplementary material for: Self-organizing pattern of subpleural alveolar ducts
Source: Sci Rep. 2020 Feb 21;10:3185. doi: 10.1038/s41598-020-59752-3 (PMC7035422; doi:10.1038/s41598-020-59752-3)
Supplement: Supplementary file 1 — Supplemental info for videos. [file 41598_2020_59752_MOESM1_ESM.pdf]

Supplementary info for following manuscript;

Self-organizing pattern of subpleural alveolar ducts

Wayne Mitzner[1] (wmitzner@jhu.edu),
Jeffrey Loube[1] (jloube1@jhu.edu,
Jarrett Venezia[1] (jvenezi4@jhu.edu),
Alan Scott[1] (ascott5@jhu.edu)

Johns Hopkins University (1)

Video 1. This is a complete sequence of confocal 84 confocal sections below the visceral pleura in an optically cleared mouse left lung. Confocal sections are spaced at 10 µm intervals.

Video 2. This is a single z-stack (1.4x1.4 mm) of 243 confocal sections below the visceral pleura in an optically cleared mouse lung. Confocal sections are spaced at 3.4 µm intervals.
